# Failure of silver nanowire transparent electrodes under current flow

**DOI:** 10.1186/1556-276X-8-235

**Published:** 2013-05-16

**Authors:** Hadi Hosseinzadeh Khaligh, Irene A Goldthorpe

**Affiliations:** 1 Department of Electrical & Computer Engineering, University of Waterloo, Waterloo, ON N2L 3G1, Canada; 2 Waterloo Institute for Nanotechnology, University of Waterloo, Waterloo, ON N2L 3G1, Canada

**Keywords:** Silver nanowires, Transparent electrode, Instability, Organic solar cell

## Abstract

Silver nanowire transparent electrodes have received much attention as a replacement for indium tin oxide, particularly in organic solar cells. In this paper, we show that when silver nanowire electrodes conduct current at levels encountered in organic solar cells, the electrodes can fail in as little as 2 days. Electrode failure is caused by Joule heating which causes the nanowires to breakup and thus create an electrical discontinuity in the nanowire film. More heat is created, and thus failure occurs sooner, in more resistive electrodes and at higher current densities. Suggestions to improve the stability of silver nanowire electrodes are given.

## Background

Transparent electrodes are a necessary component in a number of devices such as touch screens, liquid crystal displays, and organic light-emitting diodes. The most commonly used transparent conductor, indium tin oxide (ITO), is expensive, has limited mechanical flexibility, and requires high deposition temperatures. Recent advances in nanomaterials have generated alternatives to ITO. Of the various materials, films consisting of random networks of solution-synthesized silver nanowires have emerged as a leading candidate [1,2]. Current conducts through the nanowires while light is able to pass through the open spaces between the nanowire networks. We have synthesized the nanowire films that have transparency and conductivity values better than competing new flexible technologies (e.g., carbon nanotube films, graphene, conductive polymers) and comparable to ITO. Furthermore, the nanowire electrodes are inexpensive, flexible, and compatible with roll-to-roll deposition techniques.

In addition, silver nanowire electrodes also scatter a portion of the transmitted light [3], making these electrodes particularly attractive for use in solar cells. Indeed, there are numerous reports about the promising device characteristics of organic solar cells using silver nanowire electrodes [4,5]. Silver nanowires are known to oxidize and corrode over a period of months in air [6]; however, there are no studies on the stability of the nanowire electrodes during use (i.e., when they are conducting current). In contrast to ITO where current conducts throughout the entire area of the film, in nanowire electrodes, electronic transport occurs only through the metal wire pathways, and these nanowire pathways have diameters less than 100 nm. Because of this, although the current densities generated in organic solar cells are relatively low (on the order of 10 mA/cm^2^, with the best performing devices generating about 17 mA/cm^2^[7]), the resulting current densities in the nanowires are very high. For example, if we assume that half of the nanowires in 12 Ω/sq silver nanowire electrodes participate in current conduction, a solar cell current density of 17 mA/cm^2^ (i.e., total current divided by the total top surface area of the film) would result in an approximate current density in the nanowires of 4 × 10^4^ A/cm^2^ (i.e., current flowing through a single nanowire divided by its cross-sectional area)^a^. For comparison, this same current flowing through a 250-nm thick ITO film results in a cross-sectional current density of 10^3^ A/cm^2^, more than an order of magnitude less.

In this paper, it is shown that at current density levels incurred in organic solar cells, silver nanowire electrodes fail in a matter of days. We report how parameters such as sheet resistance and current density affect the time to failure, as well as characterize the electrodes to investigate the failure mechanism.

## Methods

Silver nanowires dispersed in ethanol, with average diameters of 90 nm and average lengths of 25 μm, were purchased from Blue Nano Inc., Charlotte, North Carolina. The nanowire solution was diluted and then dispersed on 5 cm × 4.5 cm glass substrates using the Mayer rod coating method [3,8,9]. Films of varying nanowire densities were prepared. After deposition, the films were annealed at 200°C for 30 min to fuse the overlapping nanowire junctions, which greatly reduces the sheet resistance. The sheet resistance of the films was measured by either a 4-point probe measurement system or a multimeter. The transparencies were measured with a spectrometer with an integrating sphere, with a plain glass substrate used as the reference.

Strips of copper tape were applied on two ends of each electrode. To investigate the effects of current flow through the electrodes, a direct current (DC) power supply was used to pass a constant current across the electrodes. The current was conducted until the electrodes failed, which we define as the point when the DC power supply reached its maximum of 30 V and thus could no longer maintain the constant current. The voltage across the electrodes and the surface temperature were monitored continuously throughout the experiment using computer data collection. For the temperature measurement, a flat leaf-style thermocouple was used. The electrodes were soon afterwards imaged with a scanning electron microscope (SEM), for which a thin coating of gold on the sample was required to prevent electron charging. Transmission electron microscopy (TEM) samples were prepared by mechanically rubbing the electrodes onto copper grids overlayed with ultra-thin amorphous carbon. Both bright-field images and energy dispersive spectroscopy (EDS) spectra were obtained in the TEM.

For comparison purposes, additional nanowire electrodes were prepared, but no current was passed across them. Rather, one electrode was left in air and its sheet resistance was monitored over the period of 1 year. Other electrodes were annealed in an atmospheric furnace each at various temperatures and times. These electrodes were imaged in the SEM at various stages to see how the electrode morphology evolved throughout the annealing process.

## Results and discussion

### Electrode failure measurements

**Figure 1:**
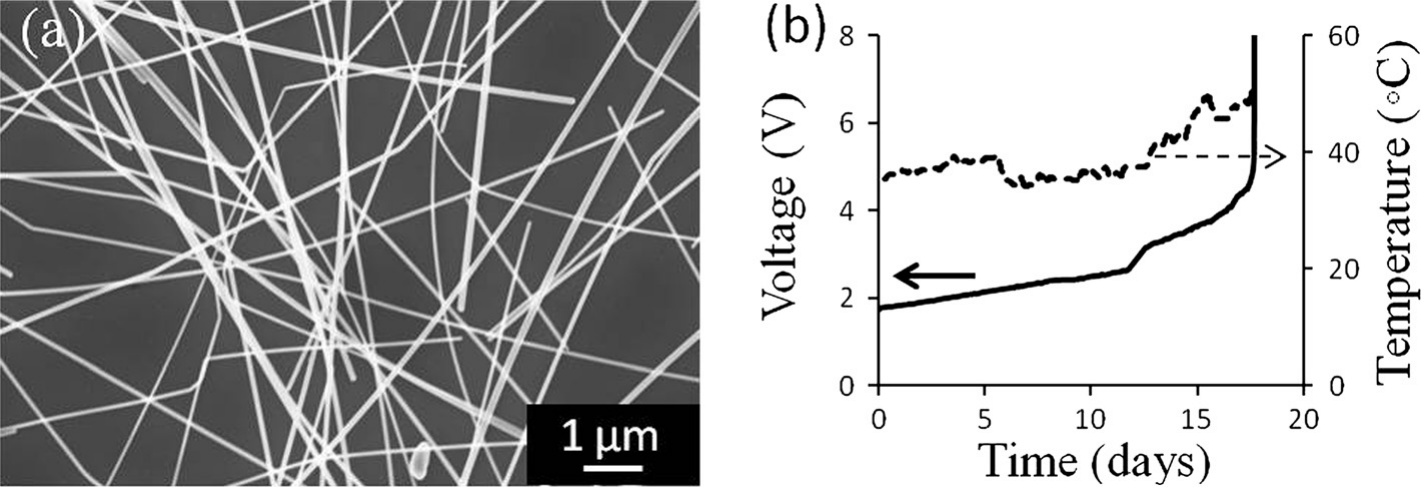
**Silver nanowire electrode and its long-term characteristics. **(**a**) SEM image of an as-prepared electrode. (**b**) Voltage and surface temperature of a 12 Ω/sq sample when a constant current density of 17 mA/cm^2^ was applied across the electrode.

An SEM image of a prepared nanowire electrode is shown in Figure 1a. The transparency of all electrodes was nearly constant across all visible wavelengths, as similarly found by other groups [3,10,11]. The electrodes prepared for the stability experiments had sheet resistances ranging from 12 Ω/sq (with a corresponding transparency of 91% at a wavelength of 550 nm) to 37 Ω/sq (with a transparency of 94% at 550 nm). Figure 1b shows the evolution of the voltage and surface temperature of a 12 Ω/sq nanowire electrode as 17 mA/cm^2^ of current was passed across it. As was typical with all samples measured, the voltage (and therefore resistance) gradually increased with time, and then suddenly jumped to 30 V once the electrode failed. The power dissipated in the electrode is *P* = *IV*, so with a constant current and a gradually increasing voltage, the surface temperature gradually increased over time as well until electrode failure.

**Figure 2:**
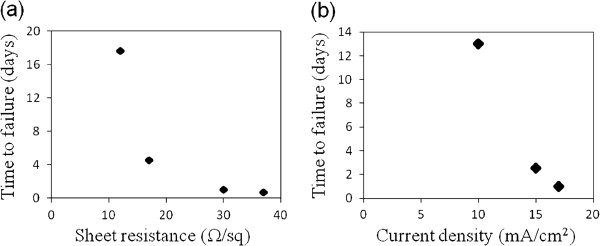
**Dependency of failure time on resistance and current density. **(**a**) The number of days to failure versus sheet resistance, when conducting 17 mA/cm^2^ across samples with different resistances. (**b**) The relationship between the number of days to failure and current density, as measured with three different 30 Ω/sq electrodes.

Figure 2a shows that under a constant current density, electrodes with a higher sheet resistance fail more quickly. Higher sheet resistance electrodes have sparser nanowire networks, and thus the current density in the individual nanowires is higher than in lower resistance electrodes. Joule heating is also higher in more resistive films, since *P* = *IV* = *I*^2^*R*. The surface temperatures immediately preceding the electrode failure of the four samples measured for Figure 2a, from the lowest to highest sheet resistance, were 55°C, 70°C, 100°C, and 102°C, respectively.

Figure 2b illustrates that for nanowire electrodes with the same sheet resistance, a higher current density results in a shorter lifetime. Higher current densities result in higher currents through the individual nanowires and more Joule heating. The temperatures of the electrode preceding failure for the three current densities applied in Figure 2b, from lowest to highest current density, were 50°C, 74°C, and 100°C, respectively.

In the comparison sample, where a nanowire electrode was left in air without current flow, the sheet resistance only increased by 10% after 3 months. After 1 year, however, the resistance was 6 orders of magnitude higher than its original value.

### Failure mechanism characterization

**Figure 3:**
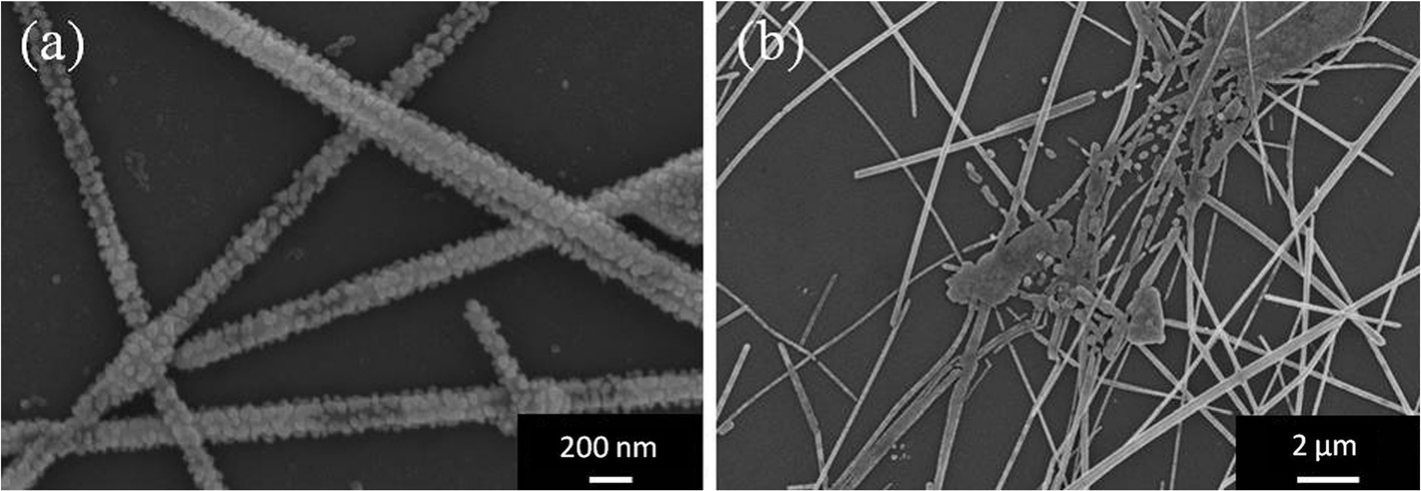
**Images of electrodes after failure. **(**a** and **b**) SEM images of a 12 Ω/sq silver nanowire electrode after a constant current density of 17 mA/cm^2 ^was passed across it for 17 days.

Typical SEM images of the electrode after failure are shown in Figure 3. In contrast to the smooth nanowire sidewalls observed in the as-prepared films, nanoparticles were now present on the nanowire surfaces. In some locations on the sample, as in Figure 3b, the nanowires were broken up into discontinuous segments. Enough nanowires in the electrode were broken up such that there was no longer a continuous electrical pathway across the film.

**Figure 4:**
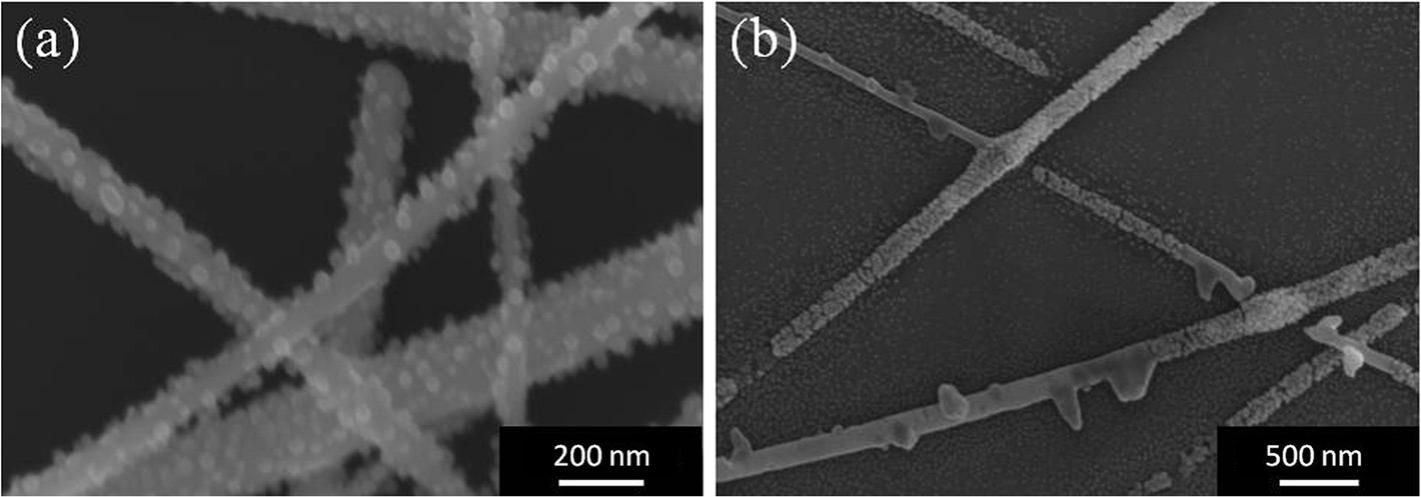
**Images of electrodes after annealing. **SEM images of silver nanowire electrodes annealed for 17 days (**a**) at 100°C and (**b**) at 150°C.

Although silver is susceptible to electromigration at the current densities and temperatures encountered in these electrodes [12], the SEM images are not indicative of the voids and hillocks that are characteristic of electromigration [12-16]. Rather, our study suggests that it is the instability of nanowires at elevated temperatures which is the reason for the electrode failure. As mentioned in the experimental section, nanowire electrodes were annealed at various temperatures without current flow. Figure 4 shows SEM images of nanowire electrodes annealed for 17 days at 100°C and 150°C. Even at a temperature as low as 100°C, nanoparticles formed on the surfaces of the nanowires (Figure 4a), which increased in size and density with increasing annealing time. At 150°C, nanoparticles also formed, and the nanowires eventually broke up into discontinuous segments (Figure 4b).

As noted in the previous section, when current is passed through a nanowire electrode, the temperature is elevated due to Joule heating. The Joule heating of silver nanowire films has been discussed previously in the context of transparent film heaters, and it was observed that this heating in some cases led to the destruction of the film [17]. Although the surface temperature of the electrodes in our studies was around or below 100°C while conducting current, the temperature of the nanowires themselves are intuitively higher than the average surface temperature, particularly at the resistive junctions where two nanowires overlap. The annealing experiments showed that nanowire networks in air at modest temperatures are unstable; nanoparticles first form and then the nanowires eventually break up and become electrically discontinuous. Thus, in the case of current conduction, the temperature of the nanowires rises due to Joule heating, and the instability of the nanowires at these temperatures causes the electrodes to fail. The measured surface temperature of the 12 Ω/sq electrode under 17 mA/cm^2^ of current flow was 55°C at the time of failure. Comparing the time to failure of this electrode to the time for the nanowires in the annealed samples to break up, we estimate that the temperature of the nanowires themselves in this particular case was between 100°C and 150°C.

**Figure 5:**
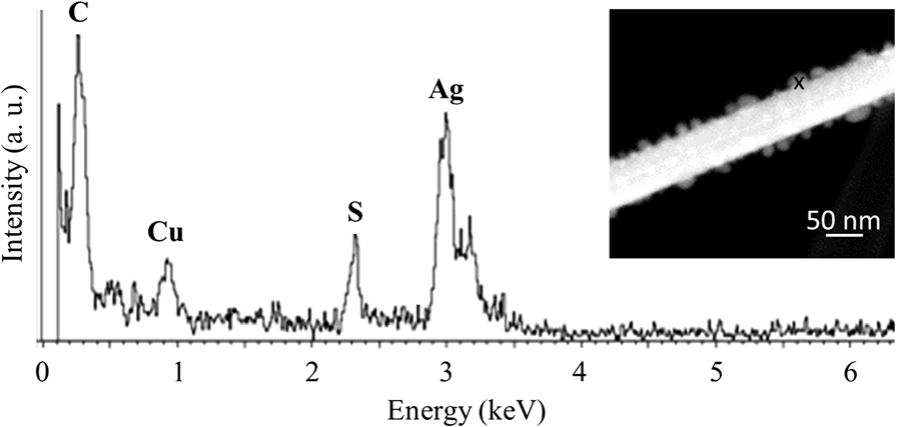
**Energy-dispersive spectrum of a nanoparticle formed on a silver nanowire after electrode failure. **The ‘x’ indicates the location where the measurement was taken. Sulfur was detected in the nanoparticles indicating corrosion of the silver.

Elechiguerra et al. found that silver nanowires synthesized by the polyol method corrode in the atmosphere [6]. Rather than corroding by reacting with oxygen, silver corrodes due to reduced sulfur gases present in the air. They observed that after 3 weeks, silver sulfide (Ag_2_S) nanoparticles started to form on the surface of the nanowires, and after 6 months, some of the nanowires became discontinuous. In our experiments, nanoparticles and breakage occur much faster. Corrosion is greatly enhanced at elevated temperatures [18]. EDS spectra were taken from the nanoparticles decorating the surface of the nanowires after electrode failure (Figure 5). Other than the carbon and copper signals originating from the TEM grid, only silver and sulfur were detected. The ratio of silver to sulfur content was 9:1. The presence of sulfur indicates that the electrodes may have failed due to the corrosion of the nanowires in the atmosphere at the elevated temperatures caused by Joule heating.

Alternatively, or addition to corrosion, another reason for the breakup of the silver nanowires at increased temperatures could be attributed to the high surface energy of the nanowires. Nanowires have a large surface-area-to-volume ratio, and the sidewalls of the nanowires used in the electrodes are all {110} planes [19], which are not the lowest energy planes in an FCC material. At elevated temperatures, atomic diffusion is increased, and kinetic limitations to reconstruction can be overcome. Silver nanobelts and nanowires of other metals have been shown to fragment at temperatures far below their bulk melting temperatures due to Rayleigh instability [20,21], and a similar phenomenon may be occurring here.

Our data indicate that the Joule heating effect elevates the temperature of silver nanowire electrodes, which leads to nanowire instability and ultimately electrode failure. More studies are required to determine whether the instability of silver nanowires at elevated temperatures in air is due to corrosion, Rayleigh instability, or another mechanism.

### Relevance to nanowire electrode design

The experimental results indicate that steps must be taken to improve the longevity of nanowire electrodes under current flow before they are suitable for use in organic solar cells. In a flexible organic solar cell, the substrate underneath the transparent electrode is typically a plastic such as polyethylene terephthalate (PET) or polyethylene naphthalate (PEN), and organic materials are deposited on top of the electrode. PET and PEN are permeable to gas [22], as are many of the common small molecules and polymeric materials used in organic solar cells [23,24], and so these materials will likely not prevent corrosion. Researchers are developing organic solar cell materials with low permeability to gas [25,26]. Alternatively, encapsulation of the organic solar cell [22,27] may prevent the corrosion of the silver nanowire electrode.

Another option is to passivate the silver nanowires. Ramasamy et al. encapsulated silver nanowires in TiO_2_[28]. The TiO_2_ shell suppressed the motion of silver atoms at the nanowire surface, thus increasing their thermal stability to 700°C. However, because of the low conductivity of TiO_2_, it is expected that the junction resistance between overlapping wires and thus the overall sheet resistance of a film of these wires would be increased significantly over bare silver nanowire films. Ahn et al. coated the surface of a silver nanowire film with graphene oxide, which is impermeable to gas molecules [29]. The coating reduced but did not completely prevent the increase of sheet resistance of silver nanowire electrodes when annealed at 70°C in high humidity over 1 week [29]. Most recently, Kim et al. sandwiched a silver nanowire electrode between two films of ZnO [30]. The composite was thermally stable up to 375°C. This ZnO passivation seems promising; however, the stability of the composite electrode at elevated temperatures for extended periods of time or its stability under sustained current flow was not reported. More study is required to develop and test a suitable silver nanowire electrode passivation.

Larger diameter nanowires would take longer to corrode and also have smaller surface-area-to-volume ratios and would thus be more stable at elevated temperatures. However, the use of larger diameter nanowires will result in less desirable optoelectronic properties (e.g., more haze, less uniformity, and potentially lower transparencies at a given sheet resistance) [31], and so there would be a trade-off between increased stability and decreased optoelectronic performance of the electrode. Another potentially helpful strategy would be to synthesize and deposit films of silver nanowires which have low energy {111} facets. Also, alternative metallic nanowires that are less susceptible to corrosion could be considered, such as cupronickel nanowires [32]. Our results also indicate the importance of keeping current densities low and using low resistance nanowire electrodes, which are unfortunately less transparent.

## Conclusions

This paper shows that at current levels generated in organic solar cells, silver nanowire electrodes fail in an unacceptably short time. Electrodes with higher sheet resistances and electrodes subject to higher current densities fail more quickly. The reason for electrode failure is attributed to the instability of silver nanowires at elevated temperatures caused by Joule heating. Design factors such as passivation, electrode sheet resistance, and nanowire diameter need to be considered before silver nanowire electrodes will be useful as an ITO replacement in organic solar cells.

## Endnotes

^a^The current density in the nanowires was estimated by dividing the total current flowing across the electrode by the total cross-sectional area of all nanowires contacting the copper strip at one end of the sample and multiplying by two since we assumed only half of the nanowires were involved in conduction.

## Abbreviations

DC: direct current; EDS: energy dispersive spectroscopy; ITO: indium tin oxide; PEN: polyethylene naphthalate; PET: polyethylene terephthalate; SEM: scanning electron microscope; TEM: transmission electron microscopy.

## Competing interests

The authors declare that they have no competing interests.

## Authors’ contributions

HHK participated in the design of the study, carried out the experiments, and drafted the manuscript. IAG supervised the project, participated in the design of the study and analysis of its results, and revised the manuscript. Both authors read and approved the final manuscript.
